# Vessel-Associated Transforming Growth Factor-Beta1 (TGF-β1) Is Increased in the Bronchial Reticular Basement Membrane in COPD and Normal Smokers

**DOI:** 10.1371/journal.pone.0039736

**Published:** 2012-06-29

**Authors:** Amir Soltani, Sukhwinder Singh Sohal, David Reid, Steve Weston, Richard Wood-Baker, E. Haydn Walters

**Affiliations:** NHMRC National Centre of Research Excellence for Chronic Respiratory Disease, School of Medicine, University of Tasmania, Hobart, Australia; Helmholtz Zentrum München/Ludwig-Maximilians-University Munich, Germany

## Abstract

**Background:**

Transforming growth factor-beta1 (TGF-β1) is a multipotential cytokine with angiogenic activity. There are only limited data about its role in airway remodeling in COPD. We have previously shown that the reticular basement membrane (Rbm) is hypervascular in the airways of current smokers either with or without chronic obstructive pulmonary disease (COPD). This study evaluated TGF-β1 immunostaining in the Rbm and its relationship to vascularity in smokers with or without COPD.

**Methodology/Principal Findings:**

Bronchial biopsies from 15 smokers with normal lung function, 19 current and 14 ex-smokers with COPD were immunostained for TGF-β1 antibody and compared to 17 healthy controls. The percentage area of tissue and also number and area of vessels staining positively for TGF-β1 were measured and compared between groups. Some bronchial biopsies from current smoking COPD subjects were also stained for phosphorylated (active) Smad2/3. Epithelial TGF- β1 staining was not different between COPD current smokers and normal controls. TGF-β1 stained vessels in the Rbm were increased in smokers with normal lung function, current smoking COPD and ex-smokers with COPD compared to controls [median (range) for number of vessels/mm Rbm 2.5 (0.0–12.7), 3.4 (0.0–8.1) and 1.0 (0.0–6.3) vs. 0.0 (0.0–7.0), p<0.05]. Percentage of vessels stained was also increased in these clinical groups. Preliminary data suggest that in current smoking COPD subjects endothelial cells and cells in the Rbm stain positively for phosphorylated Smad2/3 suggesting TGF-β1 is functionally active in this situation.

**Conclusions/Significance:**

Vessel-associated TGF-β1 activity is increased in the bronchial Rbm in smokers and especially those with COPD.

## Introduction

Chronic obstructive pulmonary disease (COPD) is a common disease involving both lung parenchyma and airways [1], [2], [3]. Cigarette smoking is the most common cause of COPD [4]. Airway structural changes, termed as airway “remodeling”, occur during the course of the disease [5], but there are only a few published papers on airway remodeling in COPD especially over recent decades [6]. Angiogenesis (vascular development and new vessel formation) is a component of remodeling in most chronic inflammatory diseases including airway diseases such as asthma [7]–[12].

Transforming growth factor-beta (TGF-β) is a multifunction cytokine with angiogenic activity which is present in many tissues and cells in the human body [12]–[18]. TGF-β1 is the most abundant isoform [19], with both structural and inflammatory cells being a source of TGF-β in the lungs [20], [21]. A previous study has shown a correlation between epithelial TGF-β and vascular endothelial growth factor (VEGF), a well-known angiogenic factor, in COPD bronchioles [22]. In addition, TGF- β receptors are suggested to play an important role in the pathogenesis of COPD through their regulation of Smad pathways [23].

We have previously reported hypervascularity of the reticular basement membrane (Rbm) of endo-bronchial biopsies (BB) from smokers with or without COPD [24]. Increased vascularity in the Rbm was associated with increased vessel-associated VEGF.

To further study angiogenic mediator expression in the BB and examine its relationship with vascular changes in the mucosa, we decided to stain our samples with anti-TGF-β1 monoclonal antibody. We hypothesized that: 1. TGF-β1 is increased in the Rbm in current smoking subjects, 2. TGF-β1 levels would positively correlate with the changes in vascularity we have previously described [24].

## Methods

### Ethics Statement

The study was approved by *Human Research Ethics Committee (Tasmania) Network.* All subjects provided written informed consent.

### Study Design

This was a cross-sectional study.

### Subjects

65 subjects were recruited through advertisement. BB from 15 smokers with normal lung function (S-N), 18 current smoking COPD (S-COPD) and 13 ex-smokers with COPD (ES-COPD) were compared with 17 healthy nonsmokers (H-N). COPD was diagnosed according to the GOLD guidelines [4]. Subjects with other respiratory diseases, a history of recent acute exacerbations of COPD and those on systemic or inhaled corticosteroids over the last 12 weeks were excluded from the study. COPD subjects were on short-acting inhaled anticholinergics only.

#### Fiberoptic bronchoscopy

Was performed as previously described [24]. There were no major complications from the procedures.

#### Pulmonary function tests

Were performed and interpreted according to ATS/ERS guidelines [25].

### Tissue Processing

Biopsies were fixed in 10% neutral buffered formalin for two hours, and then transferred to a 50% ethanol until being processed using a Leica ASP 200 tissue processor, two 3-µm paraffin embedded sections were cut for staining being separated by at least 50 microns and mounted on a slide.

After removal of paraffin, sections were stained with either monoclonal antibody anti-TGF b1 (abcam ab 27969 clone TB1 at 1/16000 −6.25×10−5 mg/ml- overnight at room temperature) after blocking with Dako serum block (X0909) or phosphorylated Smad2/3 (pSmad2/3) (Santa Cruz SC-11769R at 1/100 for 1hour at room temperature) following heat retrieval using a Dako PT link with high pH solution K800421 at 95 degrees for 30 minutes. All slides were then treated to remove endogenous peroxidase using 3% hydrogen peroxide. The primary antibody was elaborated using either anti-mouse or anti-rabbit horseradish peroxidase (HRP) conjugated DAKO Envision + reagent (K4001 or K4003) for secondary antibody binding and colour resolution using Dako DAB+ (K3468). Nuclei were counterstained using Mayers Haematoxylin and sections dehydrated through ascending grades of ethanol, cleared in xylene and mounted in permount. An equivalent isotype control using IgG1 (Dako X093101 or X090310) was run with each section and a known lung tissue positive tissue control was run with each staining.

### Measurements Slides

Were coded and randomized to blind the person who did the measurements. As many pictures as possible were taken from each slide. Only areas with intact epithelium and LP and without any holes or tissue damage were selected for photography. Overlapping areas were avoided. Then, eight areas were randomly selected for measurements. Measurements were performed using computer-assisted image analysis (Image-Pro, version 5.1, Media Cybernetics, USA) at x400 magnification.

**Figure 1:**
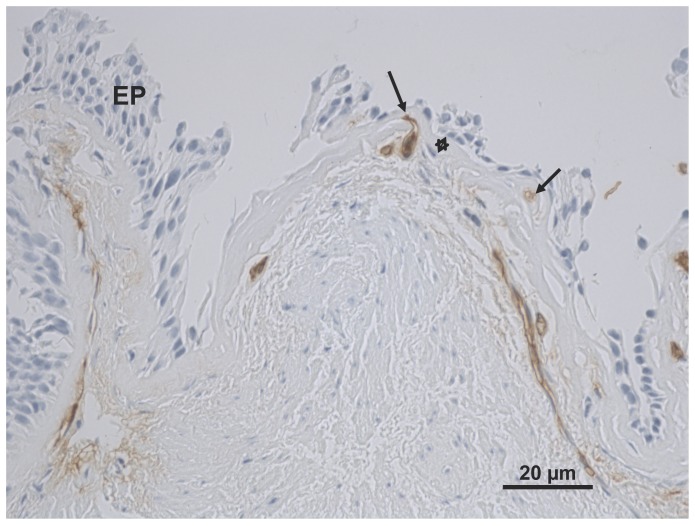
Vessels in the airway reticular basement membrane (Rbm) stained for transforming growth factor-beta1 (TGF-β1). The reticular basement membrane is indicated by a star beneath the epithelium (EP). Vessels in the Rbm are indicated by arrows. Current smoking COPD subject, X400, Scale  = 20 µm.

The percent area of tissue stained for TGF-β1 in the epithelium was measured using the automated software. The number and cross-sectional area of vessels stained by TGF-β1 in the Rbm was measured (Figure 1) and corrected for the length of the Rbm before analyses. The percentage of total vessels in the Rbm staining for TGF-β1 was calculated by dividing the number of vessels stained for TGF-β1 by the total number of vessels stained with anti-Collagen IV antibody. We used the vessel data collected in our previous published study [24] which was done in sections serially contiguous with those used currently. For TGF-β1 staining the percentage of total vessels stained for TGF-β1 was sometimes above 100% because of slight differences in the disposition of countable vessels even in serial sections. We take such data to mean in reality essentially 100% vessel staining, but the data has not been normalized for this.

45 subjects had enough analyzable tissue for final analyses.

### Statistical Analyses

Non-parametric ANOVA (Kruskal-Wallis) and post-hoc Mann-Whitney U tests were used to test mean differences amongst all study groups and between two groups respectively as the distribution of variables were non-normal. Spearman’s rank correlation coefficient was used to test for correlations between outcomes. All analyses were performed using PASW statistics 18. Two-tailed p values <0.05 were considered as significant.

## Results

Demographics of participants are summarized in Table 1. The mean age of participants with COPD was greater than the smokers with normal lung function or healthy non-smokers. The total smoking history was greater and lung function measures were lower in the COPD groups compared to smokers with normal lung function or healthy non-smokers.

**Table 1:** Demographics.

Groups* (numbers)	H-N (n = 17)	S-N(n = 15)	S-COPD (n = 19)	ES-COPD (n = 14)	P value
Age† years	54(20–68)	46(30–65)	61(46–78)	61(53–69)	<0.01
Female/male	6/11	4/11	8/11	5/9	0.2 (NS)^§^
Pack-years of smoking†	0	35(11–57)	45(18–82)	55(18–151)	<0.01
FEV1%† ^‡^	119(114–124)	100(78–125)	83(55–102)	83(55–105)	<0.01
^FEV1/FVC%^ † ^‡^	82(71–88)	78(70–96)	59(46–68)	57(38–68)	<0.01
DLCO% predicted† ml/min/mmHg	–	77(58–105)	67(48–83)	64(45–74)	<0.01

* H-N: healthy and nonsmoker, S-N: smokers with normal lung function, S-COPD and ES-COPD: current smokers and ex-smokers with COPD.

† Median (range), ^‡^ post-bronchodilator values, ^§^Chi-square test, NS  =  not significant.

As we were mainly interested in the vessel changes in the Rbm and since there was no significant difference in percent area of the epithelium stained for TGF-β1 between the “extreme groups” S-COPD and H-N groups [median (range) 6% (0–31) vs. 21% (0–51) respectively, p = 0.3] in the preliminary “scoping” study, no further measurements were performed for the other two groups.

**Figure 2:**
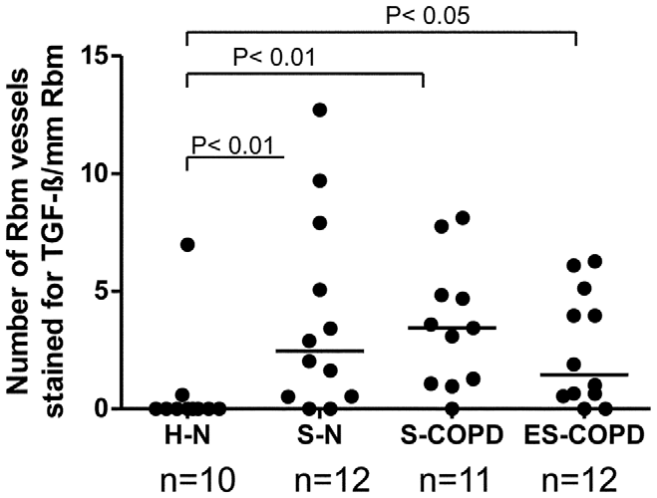
Number of vessels stained for transforming growth factor-beta1 (TGF-β1) in the reticular basement membrane (Rbm). Number of vessels are compared in healthy nonsmokers (H-N), smokers with normal lung function (S-N), current smoking COPD (S-COPD) and ex-smokers with COPD (ES-COPD).

The number and area of vessels stained for TGF-β1 in the Rbm were significantly different between groups (Kruskal-Wallis test, p<0.01 for both comparisons), with S-N, S-COPD and ES-COPD demonstrating a higher number of TGF-β1 stained vessels/mm Rbm compared to the control group [median (range) for S-N 2.5 (0.0–12.7), for S-COPD 3.4 (0.0–8.1) and for ES-COPD 1.4 (0.0–6.3) vs. H-N 0.0 (0.0–7.0)] (Figure 2). The area of vessels staining for TGF-β1, expressed per µm^2^/mm of Rbm, was also greater in S-N, S-COPD and ES-COPD [median (range) 379 (0–2132), 324 (0–2882) and 155 (0–4029)] compared to the healthy control group [median (range) 0 (0–545)], (p<0.01 for all comparisons).

**Figure 3:**
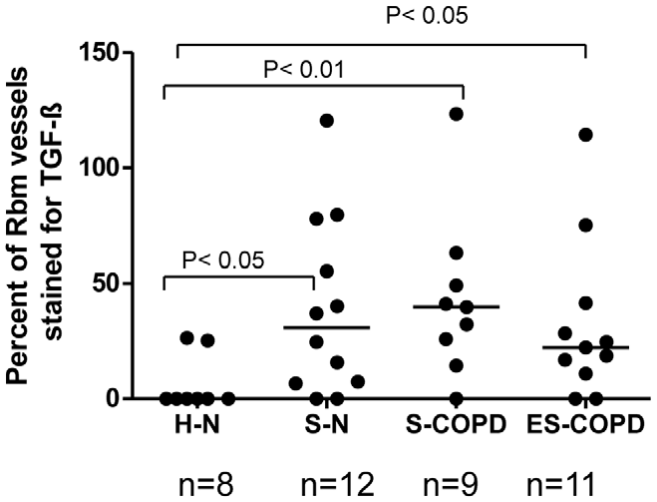
The percentage of vessels stained for transforming growth factor-beta1 (TGF-β1). The percentage is compared in healthy nonsmokers (H-N), smokers with normal lung function (S-N), current smoking COPD (S-COPD) and ex-smokers with COPD (ES-COPD). This ratio is calculated by division of the number of vessels stained for TGF-β1 by the total number of vessels in the reticular basement membrane (Rbm) marked with anti-Collagen IV antibody in serial sections, which we assessed in our previous study (Soltani A. et al., Respiratory Research. 2010, 30;11∶105). Variation in total number of vessels between sections gives occasionally anomalous data-points greater than 100%. The number of subjects in this plot is different from Figure 3 because some individuals did not have enough tissue to provide adequate consecutive sections for both types of immunostaining.

Furthermore, the percentage of total vessels in the Rbm staining for TGF-β1 was significantly different between groups (Kruskal-Wallis test, p<0.05), with S-N, S-COPD and ES-COPD having a higher percentage than the control group [median (range) for S-N 31% (0–121), for S-COPD 40% (0–123) and for ES-COPD 22% (0–114) vs. H-N 0 (0–26)] (Figure 3).

**Figure 4:**
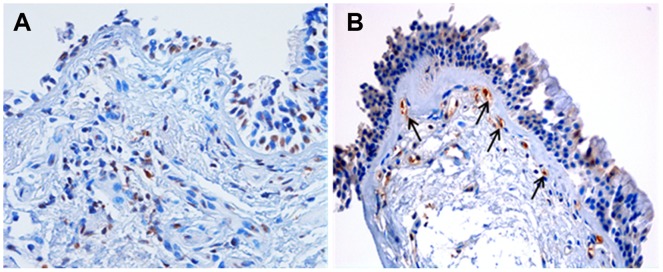
Phosphorylated (activated) Smad2/3 (pSmad2/3) staining of bronchial biopsies in normal nonsmoking controls (A) and current smoking COPD subjects (B). Black arrows indicate vessels stained with pSmad2/3 in the bronchial Rbm in current smoking COPD. There are very few vessels at all in the Rbm in normals and those that are there do not stain for pSmad2/3. X 400.

Bronchial biopsy sections from S-COPD stained positively for pSmad2/3 in the basal epithelium, cells in the Rbm, structural cells in the lamina propria, and also in vessel endothelium. Subjectively, this was strikingly more than in controls, but we do not yet have sufficient data for a full analysis (Figure 4).

### Correlations

The number of Rbm vessels stained for TGF-β1 correlated with total vessel number stained with anti-Collagen IV antibody in the ES-COPD and S-N groups, but not in the actively smoking COPD group (S-COPD) (Table 2). The total vessels staining for TGF-β1 in the Rbm correlated reasonably strongly with number of vessels stained for vascular endothelial growth factor (VEGF, measured in our previous study [24] ) only in the S-COPD group, but this did not reach statistical significance (r = 0.6, p = 0.1), but is probably a type 2 error.

**Table 2:** Correlation of total number of TGF-ß1 stained vessels against total number of vessels.

Groups	S-COPD	ES-COPD	S-N
Correlation coefficients	r = 0.1, p = 0.7	r = 0.6, **P<0.05**	r = 0.6, **P<0.05**

All correlations are Spearman’s correlation coefficients.

Abbreviations: ES-COPD, ex-smokers with COPD; Rbm, reticular basement membrane; S-COPD, Current smoking COPD; S-N, smokers with normal lung function; TGF-β1, transforming growth factor-β1.

## Discussion

This study found that in smokers with normal lung function and in subjects with COPD, airway vessel staining for TGF-β1 was increased in the Rbm. This finding could not be explained simply by TGF-β1 vessel staining reflecting gross hypervascularity of the Rbm, because the percentage of total vessels in the Rbm staining for TGF-β1 which takes this into account was also increased. Total number of vessels stained for TGF-β1 correlated with the total number of vessels in the Rbm only in the S-N and ES-COPD groups and not in the group with more vessels, the S-COPD group [24]. Thus, TGF-β1 would seem to have probably a role in vessel remodeling in both smokers and in COPD but the extra effect in S-COPD must be contributed by another factor.

There was a borderline positive correlation between total Rbm vessels staining for TGF-β1 and vessel-associated VEGF in the Rbm [24], but only in S-COPD. This might imply that in this group VEGF is the additional factor that makes the Rbm so relatively hypervascular. Thus, in smoking per se and in ES-COPD, TGF-β1 is the angiogenic driver, but in S-COPD both TGF-β1 and VEGF may be active. Epithelial TGF-β1 staining was not different between COPD current smokers and normal controls.

We observed positive staining for pSmad2/3 in the basal epithelium, cells in the Rbm, structural cells in the lamina propria and vessels in both the lamina propria and the Rbm in S-COPD. Some staining was present in normal nonsmoking controls, but subjectively was strikingly less. This suggests that TGF-β1 activity may be increased in this compartment as well as merely expression. Further work on this is ongoing.

The role of TGF-β1 in COPD pathogenesis is not clear. Some studies have shown a relationship between TGF-β1 gene single nucleotide polymorphisms and COPD development [26], but published data on TGF-β1 protein expression in the airways or lung parenchyma in COPD are both limited and contradictory. Thus, there are three studies that have published data for TGF-β1 staining in the mucosa of large airways [27], [28], [29], but they reported remarkably different results, which may reflect the differences in their criteria for subject recruitment.

Zanini et al., using BB, found higher number of cells positive for TGF-β1 in the LP in COPD subjects who were ex-smokers and not on inhaled corticosteroids and there was a correlation between cells positive for TGF-β1 and vascularity in the LP. Vessels related to the Rbm and deeper lamina propria were not differentiated. [27]. Kokturk et al. used BB to compare epithelial TGF-β1 in COPD or asthma with controls [29] and did not find any differences in epithelial and subepithelial TGF-β1 between COPD and controls. Vignola et al. also used BB to compare TGF-β1 in subjects with symptomatic chronic bronchitis (i.e. productive cough) with or without COPD, asthma and controls and found higher TGF-β1 levels in the epithelium and LP in chronic bronchitis compared to the control group [28] with no apparent relationship with COPD as such. Our data on TGF-β1 in the epithelium is similar to that of Kokturk et al. [29] but contrasts to that of Vignola et al. [28] However, Vignola et al. targeted subjects with chronic bronchitis rather than COPD, and chronic bronchitis was not one of our inclusion criteria and is not as common in Australia as Europe. Not surprisingly, chronic bronchitis was not a feature in our COPD subjects. We are the first group which has studied TGF-β1 specifically in the bronchial Rbm compartment of COPD subjects and S-N to examine its relationship to hypervascularity of the Rbm. We believe that the Rbm is a particularly pathogenic area in COPD [24], [30], and may have particular relevance to long-term outcomes such as cancer development.

A few investigators have used resected lung tissue to study TGF-β1 in the small airways and lung parenchyma in COPD [13], [31]. Clearly these studies are not fully comparable to ours which has used large airway endo-bronchial biopsies. One study found higher airway and alveolar epithelial TGF-β1 in COPD subjects without chronic bronchitis compared to controls [31], while another compared current or ex-smokers with COPD to current or ex-smokers without COPD and found higher TGF-β1 in bronchiolar and alveolar epithelium in the COPD groups [13]. The drawback for these types of studies obviously is that they cannot recruit truly healthy subjects and even their controls had undergone thoracic surgery for a clinical indication, mostly smoking-related lung cancer. Nevertheless, Takizawa et al. using an ultra-thin bronchoscope for brushing of bronchiolar epithelial cells [32] found higher epithelial TGF-β1 in smokers with normal lung function as well as COPD subjects compared to normal controls.

In general, our COPD subjects were somewhat older than the subjects in our other groups, but comprehensive regression analyses did not show any relationship between age and our positive findings, either for vessel numbers or angiogenic growth factor staining.

In conclusion, vessel-associated TGF-β1 expression was increased in both smokers and in COPD, but especially so in actively smoking COPD. Regression analysis suggests that TGF-β1 may be a driving factor for angiogenesis in these situations but that the extra effect in S-COPD is likely to be due to something else, probably VEGF. Preliminary data suggest that the TGF-β1 is indeed functionally active through pSmad2/3 expression. The Rbm may be of particularly pathological relevance in smokers and COPD [24], with the Rbm demonstrating fragmentation, hyper-vascularity and hyper-cellularity; the latter due to what we believe are migrating and transitioning epithelial cells (epithelial mesenchymal transition, EMT) [24], [30], [33]. How these cells may contribute to changes in ECM proteins, airway wall stiffness and obstruction in COPD needs to be defined. Similarly, the role of angiogenesis and EMT in smoking/COPD-related lung cancer needs further definition. Longitudinal studies to evaluate the effects of smoking cessation and also the effects of treatment, particularly inhaled corticosteroids, on TGF-β1 level in BB are also now indicated, as TGF-β1 may be a leading mediator for all these other pathological features of COPD.

## References

[1] Chapman KR, Mannino DM, Soriano JB, Vermeire PA, Buist AS (2006). Epidemiology and costs of chronic obstructive pulmonary disease.. Eur Respir J.

[2] Halbert RJ, Isonaka S, George D, Iqbal A (2003). Interpreting COPD Prevalence Estimates: What Is the True Burden of Disease?. Chest.

[3] Hogg JC (2008). Lung structure and function in COPD.. Int J Tuberc Lung Dis.

[4] GOLD (2009). Global Initiative for Chronic Obstructive Pulmonary Disease.. Global strategy for the Diagnosis, Mangement and Prevention of Chronic Obstructive Pulmonary Disease Updated 2009.

[5] Boulet LP, Sterk PJ (2007). Airway remodelling: the future.. Eur Respir J.

[6] Walters EH, Reid D, Soltani A, Ward C (2008). Angiogenesis: A potentially critical part of remodelling in chronic airway diseases?. Pharmacol Ther.

[7] Reid DW, Yudong WEN, David PJ, Trevor JW, Chris W (2008). Bronchodilator reversibility, airway eosinophilia and anti-inflammatory effects of inhaled fluticasone in COPD are not related.. Respirology.

[8] Makinde T, Murphy RF, Agrawal Devendra K (2006). Immunomodulatory Role of Vascular Endothelial Growth Factor and Angiopoietin-1 in Airway Remodeling.. Curr Mol Med.

[9] Feltis BN, Wignarajah D, Zheng L, Ward C, Reid D (2006). Increased Vascular Endothelial Growth Factor and Receptors: Relationship to Angiogenesis in Asthma.. Am J Respir Crit Care Med.

[10] Li XUN, Wilson John W (1997). Increased Vascularity of the Bronchial Mucosa in Mild Asthma.. Am J Respir Crit Care Med.

[11] Dunnill M (1960). The pathology of asthma, with special reference to changes in the bronchial mucosa.. J Clin Path.

[12] Knox AJ, Stocks J, Sutcliffe A (2005). Angiogenesis and vascular endothelial growth factor in COPD.. Thorax.

[13] de Boer Willem I, van Schadewijk A, Sont Jacob K, Sharma Hari S, Stolk JAN (1998). Transforming Growth Factor beta 1 and Recruitment of Macrophages and Mast Cells in Airways in Chronic Obstructive Pulmonary Disease.. Am J Respir Crit Care Med.

[14] Puxeddu I, Ribatti D, Crivellato E, Levi-Schaffer F (2005). Mast cells and eosinophils: A novel link between inflammation and angiogenesis in allergic diseases.. J Allergy Clinl Immunol.

[15] Jackson JR, Seed MP, Kircher CH, Willoughby DA, Winkler JD (1997). The codependence of angiogenesis and chronic inflammation.. FASEB J.

[16] Levi-Schaffer F, Pe'Er J (2001). Mast cells and angiogenesis.. Clin Exp Allergy.

[17] Roberts AB, Sporn MB, Assoian RK, Smith JM, Roche NS (1986). Transforming growth factor type beta: rapid induction of fibrosis and angiogenesis in vivo and stimulation of collagen formation in vitro.. Proc Natl Acad Sci U S A.

[18] Bertolino P, Deckers M, Lebrin F, ten Dijke P (2005). Transforming Growth Factor-Î^2^ Signal Transduction in Angiogenesis and Vascular Disorders.. Chest.

[19] Gong Y, Fan L, Wan H, Shi Y, Shi G (2011). Lack of Association Between the TGF-beta(1) Gene and Development of COPD in Asians: A Case-Control Study and Meta-analysis.. Lung.

[20] Assoian RK, Fleurdelys BE, Stevenson HC, Miller PJ, Madtes DK (1987). Expression and secretion of type beta transforming growth factor by activated human macrophages.. Proc Natl Acad Sci U S A.

[21] Magnan A, Frachon I, Rain B, Peuchmaur M, Monti G (1994). Transforming growth factor beta in normal human lung: preferential location in bronchial epithelial cells.. Thorax.

[22] Kranenburg AR, de Boer WI, Alagappan VKT, Sterk PJ, Sharma HS (2005). Enhanced bronchial expression of vascular endothelial growth factor and receptors (Flk-1 and Flt-1) in patients with chronic obstructive pulmonary disease.. Thorax.

[23] Hogg JC, Timens W (2009). The pathology of chronic obstructive pulmonary disease.. Annu Rev Pathol.

[24] Soltani A, Reid D, Sohal S, Wood-Baker R, Weston S (2010). Basement membrane and vascular remodelling in smokers and chronic obstructive pulmonary disease: a cross-sectional study.. Respir Res.

[25] Miller MR, Hankinson J, Brusasco V, Burgos F, Casaburi R (2005). Standardisation of spirometry.. Eur Respir J.

[26] Konigshoff M, Kneidinger N, Eickelberg O (2009). TGF-beta signaling in COPD: deciphering genetic and cellular susceptibilities for future therapeutic regimen.. Swiss Med Wkly.

[27] Zanini A, Chetta A, Saetta M, Baraldo S, Castagnetti C (2009). Bronchial vascular remodelling in patients with COPD and its relationship with inhaled steroid treatment.. Thorax.

[28] Vignola Antonio M, Chanez P, Chiappara G, Merendino A, Pace E (1997). Transforming Growth Factor-beta Expression in Mucosal Biopsies in Asthma and Chronic Bronchitis.. Am J Respir Crit Care Med.

[29] Kokturk N TT, Memis L, Akyurek N, Akyol G (2003). Expression of Transforming Growth Factor β1 in Bronchial Biopsies in Asthma and COPD.. J Asthma.

[30] Sohal SS, Reid D, Soltani A, Ward C, Weston S (2010). Reticular basement membrane fragmentation and potential epithelial mesenchymal transition is exaggerated in the airways of smokers with chronic obstructive pulmonary disease.. Respirology.

[31] Zandvoort A, Postma DS, Jonker MR, Noordhoek JA, Vos JTWM (2006). Altered expression of the Smad signalling pathway: implications for COPD pathogenesis.. Eur Respir J.

[32] Takizawa H, Tanaka M, Takami K, Ohtoshi T, Ito K (2001). Increased Expression of Transforming Growth Factor-{beta}1 in Small Airway Epithelium from Tobacco Smokers and Patients with Chronic Obstructive Pulmonary Disease (COPD).. Am J Respir Crit Care Med.

[33] Sohal SS, Reid D, Soltani A, Ward C, Weston S (2011). Evaluation of epithelial mesenchymal transition in patients with chronic obstructive pulmonary disease.". Respir Res.

